# Hypophosphatemia attenuates improvements in vitality after intravenous iron treatment in patients with inflammatory bowel disease

**DOI:** 10.1007/s11136-024-03642-y

**Published:** 2024-06-14

**Authors:** J. B. Bjorner, N. Kennedy, S. Lindgren, R. F. Pollock

**Affiliations:** 1 QualityMetric Incorporated LLC, Johnston, RI USA; 2 Department of Gastroenterology, Royal Devon and Exeter NHS Foundation Trust, Exeter, UK; 3 Department of Gastroenterology and Hepatology, Skåne University Hospital Malmö, Lund University, Lund, Sweden; 4 Covalence Research Ltd, Rivers Lodge, West Common, Harpenden, AL5 2JD UK

**Keywords:** Iron, Administration, Intravenous, Iron deficiency anemia, Inflammatory bowel diseases, Quality of life

## Abstract

**Purpose:**

Iron deficiency anemia is common in people with inflammatory bowel disease (IBD), causing deterioration in quality of life, which can be reversed by treatment that increases iron stores and hemoglobin levels. The present *post hoc* analyses estimate health state utility values for patients with IBD after treatment with ferric derisomaltose or ferric carboxymaltose and evaluate the health domains driving the changes.

**Methods:**

SF-36v2 responses were recorded at baseline and day 14, 35, 49, and 70 from 97 patients enrolled in the randomized, double-blind, PHOSPHARE-IBD trial (ClinicalTrials.gov ID: NCT03466983), in which patients with IBD across five European countries were randomly allocated to either ferric derisomaltose or ferric carboxymaltose. Changes in SF-36v2 scale scores and SF-6Dv2 health utility values were analyzed by mixed models.

**Results:**

In both treatment arms, SF-6Dv2 utility values and all SF-36v2 scale scores, except Bodily Pain, improved significantly (*p* =  < 0.0001). The improvement in SF-6Dv2 utility values showed no significant treatment group difference. The improvement in utility values was completely explained by improvement in Vitality scores. Vitality scores showed significantly larger improvement with ferric derisomaltose versus ferric carboxymaltose (*p* = 0.026). Patients with the smallest decrease in phosphate had significantly larger improvements in Vitality scores at each time point (*p* =  < 0.05 for all comparisons) and overall (*p* = 0.0006).

**Conclusions:**

Utility values improved significantly with intravenous iron treatment. Improvement in utility values was primarily driven by Vitality scores, which showed significantly greater improvement in the ferric derisomaltose arm. Smaller decreases in phosphate were associated with significantly higher Vitality scores, suggesting that quality of life improvement is attenuated by hypophosphatemia. The utility values can inform future cost-utility analysis.

**Supplementary Information:**

The online version contains supplementary material available at 10.1007/s11136-024-03642-y.

## Plain English summary

People with inflammatory bowel disease often have low iron levels, which can lower the levels of hemoglobin in their blood; this is called anemia. Anemia symptoms include pain and tiredness, which can interfere with the ability to perform day-to-day tasks, reducing quality of life. People with anemia and inflammatory bowel disease can be treated with iron, either orally or intravenously. Intravenous iron is used in patients with active disease, very low levels of hemoglobin, or intolerance of oral iron. A clinical study (PHOSPHARE-IBD) comparing two different formulations of intravenous iron—ferric derisomaltose and ferric carboxymaltose—found no difference in the ability of the formulations to increase hemoglobin. The choice of formulation can therefore be based on other factors, such as quality of life and frequency of adverse events. Our study aimed to compare quality of life of people treated with the different iron formulations in the PHOSPHARE-IBD study. In PHOSPHARE-IBD, quality of life was measured using a questionnaire called the SF-36v2, which includes questions on physical, social and mental health. Our study showed that quality of life improved significantly after intravenous iron, with a slightly larger improvement in patients treated with ferric derisomaltose. The biggest improvement was seen in the “vitality” section of the questionnaire, which assesses energy, fatigue, and overall vitality. Further analysis showed that the smaller improvement in quality of life after ferric carboxymaltose might be caused by low levels of phosphate, which occurs commonly after infusions of ferric carboxymaltose, but not ferric derisomaltose.

## Introduction

Anemia is a common complication in people with inflammatory bowel disease (IBD). Reported prevalence rates vary considerably among different patient populations (e.g., pediatric versus adult patients, inpatients versus outpatients), but it is estimated that overall, approximately one quarter of people with IBD have anemia [1], with iron deficiency (ID) being the most frequently occurring underlying cause [2]. ID in IBD occurs largely due to bleeding in the gastrointestinal tract, impaired iron absorption, and inadequate intake of dietary iron due to malnutrition [2].

IBD may have a profound detrimental effect on many aspects of patients’ everyday lives and can result in a lower overall quality of life (QoL) relative to the general population [3, 4]. For example, a large multinational study recently reported that over a third IBD patients felt that their IBD interfered with intimate relationships and, in terms of work, 40% of patients reported that IBD necessitated adjustments in their professional life (e.g., working part time or flexible hours) [4]. Over half of respondents believed that IBD had influenced their career choices or trajectory [4]. In addition to the disease burden itself, IBD patients who suffer from ID with or without anemia often experience symptoms such as fatigue that significantly contributes to reduced QoL and work impairment [5, 6]. Previous research has demonstrated that both hemoglobin (Hb) levels and iron status are associated with QoL in IBD [7], and that QoL improves with treatment-related increases in Hb—independent of disease activity [8]. Furthermore, IV iron has been found to improve QoL in IBD patients treated for ID either in the absence or presence of anemia [9, 10]. In general, the treatment of iron deficiency anemia (IDA) with either oral or IV iron has been shown to improve QoL [11].

The European Crohn’s and Colitis Organization (ECCO) guidelines recommend iron supplementation for all persons with IBD and IDA with the goal of normalizing Hb and restoring iron levels. Oral iron supplementation is recommended for those with mild anemia and clinically inactive disease. Intravenous (IV) iron formulations are the recommended first-line treatment for patients with clinically active disease, patients with intolerance to oral iron, those requiring erythropoiesis-stimulating agents, and those with Hb levels < 10 g/dL [12]. The preference for IV iron is based on evidence demonstrating that IV iron is more efficacious than oral iron in terms of the proportion of patients who achieve improvements in Hb levels of ≥ 2.0 g/dL from baseline, as well as a better tolerability profile, in particular in the avoidance of gastrointestinal adverse events with IV formulations [13]. Several different IV iron formulations are available for the treatment of IDA, and direct comparison of two high dose formulations (ferric derisomaltose [FDI] and ferric carboxymaltose [FCM]) showed no significant difference between the two formulations in terms of improvements in Hb levels [14]. Consequently, treatment decisions pertaining to which IV formulation to use are frequently based on other factors such as the tolerability and adverse event profile [15], dosing, healthcare resource utilization, and cost [16, 17].

Hypersensitivity reactions may occur after IV iron treatment, but modern formulations are usually well tolerated and have a similar low incidence of serious or severe hypersensitivity reactions [18]. Some IV iron formulations, however, are associated with a heightened risk of incident hypophosphatemia, notably FCM [16]. FCM-induced hypophosphatemia can be both severe and persistent [19], which can have debilitating consequences on muscle and bone health, including myopathy, osteomalacia, and fractures [20]. In the acute setting, hypophosphatemia following IV iron treatment can lead to symptoms of fatigue, bone pain, and muscular weakness [18]. In the PHOSPHARE-IBD trial, a double-blind randomized controlled trial comparing FDI and FCM at equivalent dosing, FCM treatment led to a lesser improvement in fatigue scores compared with FDI, and reduction in serum phosphate was correlated with reduced improvement in fatigue [18].

While the primary endpoint of the PHOSPHARE-IBD trial was incidence of hypophosphatemia, QoL was also reported. QoL was measured using the SF-36v2, from which SF-6Dv2 health state utility values (HSUVs) can be derived for use in health economic analyses.

The aims of this *post hoc* analysis of the PHOSPHARE-IBD trial were to estimate SF-6Dv2 HSUVs for patients with IBD and IDA after treatment with FDI or FCM, to evaluate the health domains in SF-36v2 driving the changes in SF-6Dv2 HSUVs, and to evaluate the association between reduction in serum phosphate and QoL.

## Methods

### Patient population

PHOSHARE-IBD was a double-blind trial that included adults with IBD and IDA (hemoglobin [Hb] < 13 g/dL; s-ferritin ≤ 100 ng/mL) who were unsuitable for oral iron treatment. Inclusion criteria also contained body weight ≥ 50 kg, estimated glomerular filtration rate ≥ 65 mL/min/1.73 m^2^ and serum phosphate > 2.5 mg/dL. Exclusion criteria included anemia predominantly due to factors other than IDA, hemochromatosis or other iron storage disorder, or intravenous iron use within 30 days prior to screening. Patients were also excluded if they had a known hypersensitivity to any component in FDI or FCM or had experienced a previous serious hypersensitivity reaction to any intravenous iron compound. Finally, patients weighing less than 70 kg with Hb ≥ 10 g/dL were excluded to include only patients with a minimum iron need of 1,500 mg. A total of 97 patients were recruited at outpatient clinics in 5 European countries with high prevalence and morbidity of IBD (Austria, Denmark, Germany, Sweden, and the UK) [14, 21]. Patients were randomly allocated to treatment; 48 received FDI and 49 received FCM. The sample size calculation for the trial was based on an assumed hypophosphatemia incidence rate of 15% in the FDI arm and 40% in the FCM arm, and the ability to detect significance with an alpha of 0.05 and 80% power [14]. Baseline characteristics were similar between treatment groups (Table 1). In the overall study population, mean age was 42.1 years; 52.6% of patients were female; 39.2% had Crohn’s disease; and 60.8% had ulcerative colitis [14].

**Table 1:** Baseline demographics and characteristics of patients enrolled in the PHOSPHARE-IBD trial

Characteristic	FDI	FCM
Mean (SD) age, years	42.3 (14.1)	41.9 (14.7)
Female, *n* (%)	26 (54.2)	25 (51.0)
*IBD diagnosis, n (%)*		
Crohn’s disease	16 (33.3)	22 (44.9)
Ulcerative colitis	32 (66.7)	27 (55.1)
Mean (SD) C-reactive protein, mg/L	9.5 (13.5)	13.3 (30.8)
Mean (SD) Hb, g/dL	10.5 (1.5)	10.4 (1.4)
Mean (SD) s-Ferritin, ng/mL	9.5 (9.6)	14.6 (28.7)

*FCM* Ferric carboxymaltose, *FDI* Ferric derisomaltose, *Hb* Hemoglobin, *IBD* Inflammatory bowel disease, *SD* Standard deviation

### Study procedures

Patients were randomized 1:1 to FDI or FCM (1000 mg at baseline and 500 or 1000 mg at Week 5 based on weight and iron need at baseline). The primary endpoint was the incidence of hypophosphatemia (defined as serum phosphate < 2 mg/dL at any time point between baseline and day 35). Additional endpoints included biomarkers of bone and mineral metabolism, Hb concentrations, fatigue score, QoL and adverse events. QoL was measured using the SF-36v2 at baseline (day 0) and day 14, 35, 49, and 70. Further details on the study design have been reported previously by Zoller et al. [14].

### Measurement of QoL and derivation of utility values

The SF-36v2 health survey is a short-form health survey that is widely used and extensively validated. The SF-36v2 measures eight health domains: Physical Functioning, Role Physical, Bodily Pain, General Health, Vitality, Social Functioning, Role Emotional, and Mental Health. The scales were scored according to the developers’ instructions to derive scale scores with a mean of 50 and a standard deviation of 10 in the US general population and higher scores indicating better health [22]. We hypothesized that health change would be seen for the Vitality domain in particular, similar to previously reported FACIT Fatigue scale results from the PHOSPHARE-IBD trial [14]. The Vitality score is based on four questions in the SF-36v2: “Did you feel full of life?”, “Did you have a lot of energy?”, “Did you feel worn out?”, and “Did you feel tired?”.

SF-6Dv2 HSUVs were derived from ten items in the SF-36v2 through a standardized scoring procedure [23]. Responses from these ten items were combined to form six dimensions of health (Physical Functioning, Role Limitations, Social Functioning, Pain, Mental Health and Vitality), with each dimension containing 5–6 levels of health. These health levels were weighted using country-specific utility weights to derive SF-6Dv2 HSUVs. In this instance, country-specific weights from the United Kingdom were used, on the grounds that the UK has the highest prevalence of IBD in Europe [21, 24]. The final HSUV score has a maximum of 1 which represents full health. A score of 0 is equivalent to being dead, but negative scores are possible, indicating states regarded as worse than death.

Data analysis used a mixed linear model with restricted maximum likelihood estimation. A spatial power covariance structure was used based on the number of days between assessments. In light of the randomization, a common mean baseline score was estimated across the two treatment arms. Model fit evaluation was based on scaled residuals.

To evaluate the main drivers of the change in SF-6Dv2 HSUVs over time, we conducted additional analyses with SF-6Dv2 as dependent variable and including each SF-36v2 health domain scale as an explanatory variable. These analyses were pooled across the FDI and FCM arms. If the change in SF-6Dv2 score over time was eradicated by including a particular health domain scale, this scale was seen as a significant driver of the change in HSUVs.

Further, to explore the relation between serum phosphate and QoL, a pooled analysis for FDI and FCM was conducted evaluating changes in Vitality scores over time, with quartiles of the average phosphate concentration change used as the explanatory variable. Additionally, the relation was explored using linear regression analysis.

## Results

**Fig. 1:**
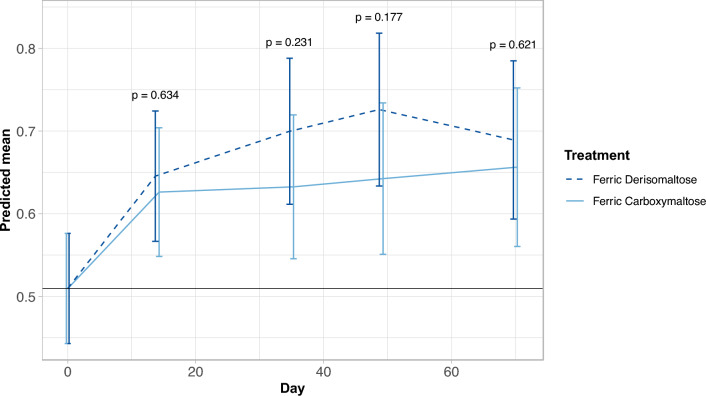
Changes in SF-6Dv2 scores over time by IV iron treatment. Estimated mean score and 95% confidence intervals. *P* values calculated based on the comparison between treatment groups in the change from baseline to each follow-up

At baseline, the mean SF-6Dv2 score for patients in the FCM arm was 0.059 points higher relative to the FDI arm (data not shown). The scores improved significantly over time in both treatment arms, with the greatest increase occurring in the first 2 weeks of treatment (Fig. 1 and Supplementary Table 1). The magnitude of improvement observed in the FDI arm was larger than the change in the FCM arm (Fig. 1). In the FDI arm, the estimated increases in SF-6Dv2 scores from baseline were 0.14 points at day 14 (*p* =  < 0.0001), 0.19 points at day 35 (*p* =  < 0.0001), 0.22 points at day 49 (*p* =  < 0.0001), and 0.18 points at Day 70 (*p* = 0.0006, Web appendix). In the FCM arm, the estimated increases from baseline in SF-6Dv2 scores were 0.12 points at day 14 (*p* =  < 0.0001), 0.12 points at day 35 (*p* = 0.003), 0.13 points at day 49 (*p* = 0.005), and 0.15 points at day 70 (*p* = 0.005, Supplementary Table 1). However, the differences in score improvements between FDI and FCM did not achieve statistical significance at any time point (Fig. 1), and test across all follow-up time points showed no statistically significant difference between the two treatment arms.

All scale scores, except Bodily Pain (*p* = 0.172), improved significantly over time for both treatment arms (*p* =  < 0.0001 for all other scales) (Supplementary Table 2). The largest improvement was typically seen at day 49, where the improvement ranged from 4.4 points (Physical Function) to 14.8 points (Vitality, Web appendix). While the scale score improvement was usually larger for the FDI arm than for the FCM arm, significant overall differences in score improvements between the treatment arms were only found for the Vitality score (*p* = 0.026) and the Role Emotional scale (*p* = 0.028) (Supplementary Table 2). However, for the Role Emotional scale, the plot of predicted mean values did not support a consistent benefit for the FDI arm compared to the FCM arm (Supplementary Fig. 2).

**Fig. 2:**
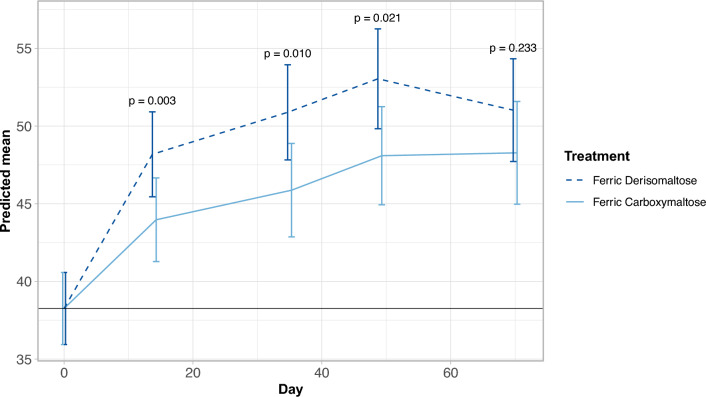
Changes in SF-36v2 Vitality scores over time by IV iron treatment—estimated mean score and 95% confidence interval. Estimated mean score and 95% confidence intervals. *P* values calculated based on the comparison between treatment groups in the change from baseline to each follow-up

Vitality scores in both treatment arms improved over time up to day 49, with larger estimated improvements in the FDI arm than in the FCM arm (day 14: 9.9 vs. 5.7 points, *p* = 0.0026; day 35: 12.6 vs. 7.6 points, *p* = 0.010; and day 49: 14.8 vs. 9.8 points, *p* = 0.021; Fig. 2 and Supplementary Table 3). From day 49 to day 70, the scores declined slightly in the FDI arm and were virtually unchanged in the FCM arm (overall improvements from baseline at day 70 were 12.7 points in the FDI arm versus 10.0 points in the FCM arm, *p* = 0.233; Fig. 2 and Supplementary Table 3).

**Fig. 3:**
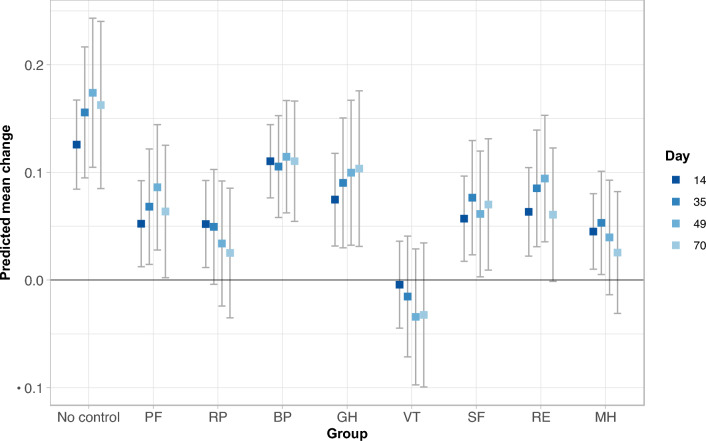
Improvement in SF-6Dv2 score from baseline across the two treatment arms, without and with control for individual SF-36v2 health domain scales. Estimate and 95% confidence interval. No control: estimated SF-6Dv2 score improvement from baseline at 14, 35, 49, and 70 days of follow-up. PF through MH: estimated SF-6Dv2 score improvement from baseline at 14, 35, 49, and 70 days of follow-up with control for each of the eight corresponding SF-36v2 health domain scales; PF: Physical Functioning; RP: Role Physical; BP: Bodily Pain; GH: General Health; VT: Vitality; SF: Social Functioning; RE: Role Emotional; MH: Mental Health

In a pooled analysis across the FDI and FCM arms, the estimated SF-6Dv2 score improvement from baseline was 0.13 (95% confidence interval [CI] 0.08 to 0.17) at day 14, 0.16 (0.09–0.22) at day 35, 0.17 (0.10–0.24) at day 49, and 0.16 (0.08–0.24) at day 70 (Fig. 3). Controlling for each individual SF-36v2 health domain scale (Physical Functioning, Role Physical, Bodily Pain, General Health, Vitality, Social Functioning, Role Emotional, and Mental Health) diminished this improvement to varying degrees (Fig. 3). The Vitality score had the most profound effect with a near complete removal of the improvement in SF-6Dv2 score (estimated [95% CI] improvement from baseline of 0.00 [− 0.04 to 0.04] at day 14, − 0.02 [− 0.07 to 0.04] at day 35, − 0.03 [− 0.10 to 0.03] at day 49, and − 0.03 [− 0.10 to 0.03] at day 70, Fig. 3). This suggests that the Vitality score is the main driver of the improvement in SF-6Dv2 score.

**Fig. 4:**
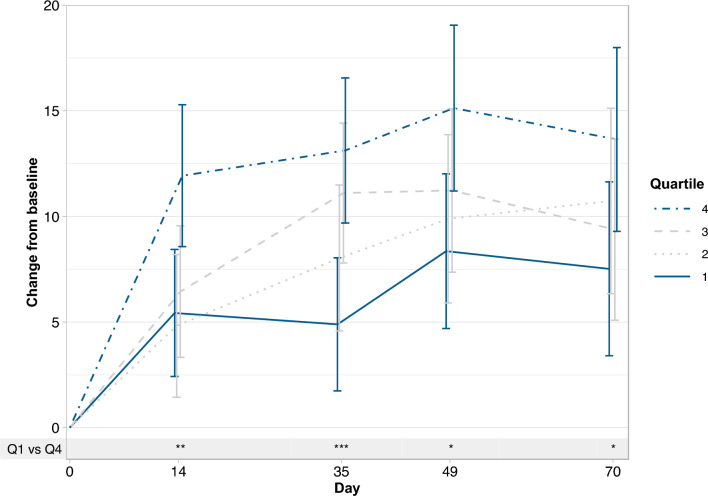
Least squares mean change (95% CI) from baseline in SF-36v2 Vitality score by s-phosphate mean change quartiles (safety analysis set). Estimates from mixed model for repeated measures with phosphate change group (quartiles), day, and stratum as factors, day-by-phosphate change group and day-by-baseline value interactions, and baseline value as covariate. For each patient, the average change from baseline phosphate was calculated using only observed values and ignoring number of post-baseline measurements. Between-quartile values indicated: *p < 0.05; **p = 0.001–0.01; ***p < 0.001

The pooled FDI-FCM analysis of changes in Vitality scale score over time and split by quartiles of the average phosphate concentration change showed that patients with the greatest decrease in phosphate (quartile 1) had the lowest increase in Vitality scores, while patients with the lowest decrease in phosphate (quartile 4) experienced the largest improvement in Vitality scores. The differences between quartile 1 and quartile 4 were significant at all time points (day 14; *p* =  < 0.01, day 35; *p* =  < 0.001 and day 49/70; *p* =  < 0.05) (Fig. 4). A linear regression analysis showed that the improvement in Vitality scale score was inversely associated with the decrease in phosphate concentration (*p* = 0.0006) (Supplementary Fig. 1).

## Discussion

In this study of HSUVs for patients with IBD after treatment with FDI or FCM, the SF-6Dv2 HSUVs showed significant improvements over time in both treatment arms. The improvement in the FDI arm was greater than the change in the FCM arm, but the difference was not statistically significant.

The improvement in both arms coincides with improvement in five of the six domains that contribute to the SF-6Dv2: Physical Functioning, Role Function (including Role Physical and Role Emotional), Vitality, Social Functioning, and Mental Health. Only the Bodily Pain scale did not show statistically significant improvement over time. While all other domain score improvements were statistically significant from baseline, the improvement in Vitality score was numerically larger than improvements in any other domains, and it was the main driver for the observed improvement in SF-6Dv2 HSUVs.

The overall improvement in Vitality score was significantly greater in the FDI arm than in the FCM arm, presumably reflecting reduced fatigue in the FDI arm. Significant differences in estimated Vitality score changes were seen between the treatment arms at day 14, 35, and 49. At day 35 and 49, the treatment arm differences were around 5 points, a magnitude normally considered clinically significant [18]. Vitality was the only scale showing significant and consistent differences between treatment arms. Since the Vitality domain is only one component of the SF-6Dv2, the treatment arm differences in this domain were not enough to introduce significant treatment arm differences in the overall SF-6Dv2 score.

To explore the underlying cause of the differences in Vitality score, a pooled analysis for FDI and FCM was conducted showing that patients with the greatest decrease in phosphate experienced the lowest increase in Vitality scores. This result was supported by a linear regression analysis showing that improvement in Vitality scale score was inversely associated with the decrease in phosphate concentration. A similar approach was used by Zoller et al. to analyze the FACIT-fatigue QoL-data from the PHOSPHARE-IBD trial, and the results from the current analysis thus support their previous finding that reduction in serum phosphate partially undermines the beneficial effect of IV iron on fatigue. Indeed, previous studies of FCM have reported fatigue persisting in the presence of hypophosphatemia, despite correction of anemia [25].

The precise mechanisms for the effects of phosphate concentration on vitality and fatigue improvements are unclear, but phosphate homeostasis is essential for many fundamental biological processes, including skeletal mineralization, energy metabolism, enzyme function, cell membrane integrity, and neurologic function [26]. These collectively help sustain a healthy physiology [27], and several case reports of hypophosphatemia following FCM treatment describe symptoms of fatigue, weakness, asthenia, muscle pain, and bone complications [18].

It is widely recognized that the impact of IBD extends beyond the objective measures of disease activity and severity and encompasses factors such as worry and anxiety, fatigue, and impaired productivity [28], which can have a substantial influence on the everyday lives of patients. Moreover, the severity and subsequent impact of more subjective measures such as fatigue and anxiety may sometimes be underestimated by healthcare providers [29]. Measures of patient-reported outcomes such as QoL therefore provide pertinent information beyond that which can be provided by objective measures of disease activity alone and are increasingly recognized as important trial endpoints [30, 31].

With regard to anemia specifically, in addition to the substantial impact on the everyday life of patients, anemia is also associated with a considerable economic burden. In a recent US-based analysis, annual healthcare costs for IBD patients with anemia were 8% higher than for those without anemia [32]. Both the direct medical costs and QoL implications of anemia are fundamental considerations for cost-effectiveness analyses in the treatment of IDA. The translation of QoL into HSUVs for different health states underlies the calculation of any incremental gain in quality-adjusted life years (QALYs) reported in cost-utility analyses. Moreover, the SF-6D is one of the most widely used and extensively validated measures for eliciting utility values and is among the most frequently recommended utility measures in pharmacoeconomic guidelines [33].

A key caveat of the SF-36 and SF-6D is that as generic measures that do not focus on disease-specific symptoms (e.g., abdominal pain/bloating, rectal bleeding) they may lack the sensitivity to detect small, yet meaningful, changes in some disease-specific domains. As such, within clinical trials the use of disease-specific measures alongside generic measures may provide a more nuanced and sensitive impression of QoL. However, in addition to facilitating health economic analyses, one of the key advantages of the use of generic measures is that it facilitates the comparability of QoL and cost-effectiveness across different disease areas, which may provide valuable information for payers faced with making systemwide reimbursement decisions based on finite budgetary constraints.

The present analysis has several limitations that should be acknowledged. In particular, the study was powered for the primary endpoint of incidence of hypophosphatemia in the main trial, and not for the QoL endpoints reported in this exploratory *post hoc* analysis. The enrollment in PHOSPHARE-IBD was relatively small (*n* = 97 patients), which may limit the robustness of the data, specifically with regard to any subgroup analyses (e.g., in patients with ulcerative colitis versus Crohn’s disease). Furthermore, while several comparisons were performed as part of the present analysis, no multiplicity correction was applied, increasing the possibility of incorrectly rejecting the null hypothesis (type I error). The correlations between the SF-36v2 scales analyzed would render traditional multiplicity correction approaches such as the Bonferroni, Hochberg, or Šidák procedures overly conservative [34–37]. While adjustments for correlated endpoints are possible using approaches such as those proposed by James [38], establishing an adjustment that reduces the type I error rate sufficiently while avoiding unnecessary loss of power is further complicated where outcome distributions are not jointly normal. Given the complexities involved and the exploratory nature of the analyses, such adjustments were not applied, and the analyses should therefore be interpreted with the potential for a type I error in mind.

For the SF-6Dv2 and for most SF-36v2 scales, baseline score differences were found between the FCM and FDI arms. Since the data stem from a double-blind randomized trial, the baseline differences must be ascribed to statistical fluctuations. That the differences are seen across many scales is likely due to the high covariance between the scale scores. Given the baseline differences between the FCM and FDI arms, the current analyses took a conservative approach by estimating a common baseline score across the two treatment arms. This reduced the estimated treatment benefits of the FDI arm but protected against a potential risk of bias due to a regression to the mean effect at the follow-up assessments.

By way of recommendations for potential future research in the area, further clinical studies could be conducted to establish if the finding of a correlation between smaller reductions in phosphate and higher Vitality scores can be confirmed in other patient populations with IBD and IDA, and if the correlation also exists in patients with other etiologies of IDA. Furthermore, more sophisticated modeling techniques, such as latent growth or structural equation models, could be employed to further explore and characterize the causal nature of the relationship between reductions in phosphate and lower SF36-v2 Vitality scores.

## Conclusions

The analysis presented here is the first to report HSUVs for IBD patients with IDA undergoing treatment with either FCM or FDI in a randomized controlled trial setting. The results showed that HSUVs improved significantly in both treatment arms, with larger improvements in the FDI arm. The Vitality score in SF36-v2 was the main driver for the observed improvement in HSUVs and the score in this domain improved significantly more in the FDI group than in the FCM group. Patients with the smallest decrease in phosphate had significantly higher Vitality scores, suggesting that the larger improvement in HSUVs in the FDI group could be caused by the higher rate of hypophosphatemia in the FCM group.

### Supplementary Information

Below is the link to the electronic supplementary material.

Supplementary file1 (DOCX 50 kb)

## Data Availability

The data supporting the findings of this study are available from the corresponding author upon reasonable request.
