# Supplementary material for: Hypophosphatemia attenuates improvements in vitality after intravenous iron treatment in patients with inflammatory bowel disease
Source: Qual Life Res. 2024 Jun 14;33(8):2285–94. doi: 10.1007/s11136-024-03642-y (PMC11286717; doi:10.1007/s11136-024-03642-y)
Supplement: Supplementary file 1 — Supplementary file1 (DOCX 50 kb) [file 11136_2024_3642_MOESM1_ESM.docx]

**Hypophosphatemia Attenuates Improvements in Vitality after Intravenous Iron Treatment in Patients with Inflammatory Bowel Disease**

**Supplementary Materials**

| **Authors** | Bjorner JB^1^, Kennedy N^2^, Lindgren S^3^, Pollock RF^4^ |
| --- | --- |
| **Affiliations** | ^1^ QualityMetric Incorporated LLC, Johnston, RI, USA  ^2^ Department of Gastroenterology, Royal Devon and Exeter NHS Foundation Trust, Exeter, UK  ^3^ Department of Gastroenterology and Hepatology, Skåne University Hospital Malmö, Lund University, Lund, Sweden  ^4^ Covalence Research Ltd, Harpenden, UK |
| **Corresponding author** | Richard Pollock Covalence Research Ltd Rivers Lodge, West Common Harpenden, AL5 2JD United Kingdom  Phone: +44 (0) 20 8638 6525 E-mail: [pollock@covalence-research.com](mailto:pollock@covalence-research.com) |

SUPPLEMENTARY MATERIALS

**Supplementary Table 1 Changes in SF-6Dv2 scores by IV iron treatment and day, and between IV iron treatments**

|  | **FDI** | | **FCM** | | **Change in score with FDI versus FCM** | | |
| --- | --- | --- | --- | --- | --- | --- | --- |
| **Day** | **Predicted mean** | **SE** | **Predicted mean** | **SE** | **Estimate** | **SE** | **p** |
| 0^1^ | 0.510 | 0.034 | 0.510 | 0.034 |  |  |  |
| 14 | 0.645 | 0.040 | 0.626 | 0.040 | 0.019 | 0.040 | 0.634 |
| 35 | 0.700 | 0.045 | 0.632 | 0.044 | 0.067 | 0.056 | 0.231 |
| 49 | 0.726 | 0.047 | 0.642 | 0.047 | 0.084 | 0.062 | 0.177 |
| 70 | 0.689 | 0.049 | 0.656 | 0.049 | 0.033 | 0.067 | 0.621 |

^1^ Estimated common baseline score across the two treatment arms; FCM: ferric carboxymaltose; FDI: ferric derisomaltose; SE: standard error. Predicted mean:

**Supplementary Table 2 Significance tests for SF-36v2 scale score improvements over time and IV iron treatment arm differences in score improvement**

|  | **Score improvements over time across treatment arms  (Day)** | **Treatment arm differences in score improvement** |
| --- | --- | --- |
| Physical Functioning | <.0001 | 0.061 |
| Role Physical | <.0001 | 0.330 |
| Bodily Pain | 0.172 | 0.555 |
| General Health | <.0001 | 0.560 |
| Vitality | <.0001 | 0.026 |
| Social Functioning | <.0001 | 0.183 |
| Role Emotional | <.0001 | 0.028 |
| Mental Health | <.0001 | 0.409 |

**Supplementary Table 3 Changes in SF-36v2 Vitality scores by IV iron treatment and day, and between IV iron treatments**

|  | **FDI** | | **FCM** | | **Change in score with FDI versus FCM** | | |
| --- | --- | --- | --- | --- | --- | --- | --- |
| **Day** | **Predicted mean** | **SE** | **Predicted mean** | **SE** | **Estimate** | **SE** | **p** |
| 0^1^ | 38.26 | 1.18 | 38.26 | 1.18 |  |  |  |
| 14 | 48.18 | 1.39 | 43.97 | 1.37 | 4.21 | 1.39 | 0.003 |
| 35 | 50.88 | 1.56 | 45.87 | 1.53 | 5.01 | 1.93 | 0.010 |
| 49 | 53.04 | 1.63 | 48.09 | 1.61 | 4.95 | 2.13 | 0.021 |
| 70 | 51.02 | 1.68 | 48.27 | 1.68 | 2.74 | 2.30 | 0.233 |

**Supplementary Figure 1 Average change in SF-36v2 Vitality score versus average change in phosphate**

**Supplementary Figure 2 Change in SF-36v2 Role Emotional score by treatment – estimated mean score and 95% confidence interval**
